# Stage-Associated Physicochemical and Chemical Profiles of a Single Production Run of *Tithonia diversifolia* Honey Mead: An Exploratory Study

**DOI:** 10.3390/foods15173048

**Published:** 2026-08-28

**Authors:** Sahutchai Inwongwan, Gerry Renaldi, Patcharin Phokasem, Chainarong Sinpoo, Tanyawat Keaewsalud, Hlaing Min Oo, Hien Thu Aung, Jeehye Sung, Rajnibhas Sukeaw Samakradhamrongthai, Terd Disayathanoowat

**Affiliations:** 1 Department of Biology, Faculty of Science, Chiang Mai University, Chiang Mai 50200, Thailand; sahutchai.inwongwan@cmu.ac.th (S.I.); patcharin.ph@cmu.ac.th (P.P.); chainarong.s@cmu.ac.th (C.S.); 2 Research Center of Deep Technology in Beekeeping and Bee Products for Sustainable Development Goals (SMART BEE SDGs), Chiang Mai University, Chiang Mai 50200, Thailand; tanyawant095@gmail.com; 3 Center of Excellence in Microbial Diversity and Sustainable Utilization, Faculty of Science, Chiang Mai University, Chiang Mai 50200, Thailand; 4 Greater Mekong Subregion (GMS) Biodiversity and Innovation Research Center, Chiang Mai University, Chiang Mai 50200, Thailand; 5 Division of Food Science and Technology, Faculty of Agro-Industry, Chiang Mai University, Chiang Mai 50100, Thailand; gerry_ren@cmu.ac.th; 6 Division of Product Development Technology, Faculty of Agro-Industry, Chiang Mai University, Chiang Mai 50100, Thailand; 7 Interdisciplinary and Food Product Development for Wellness Research Unit (INFRU), Chiang Mai University, Chiang Mai 50100, Thailand; 8 Faculty of Pharmacy, Chiang Mai University, Chiang Mai 50200, Thailand; 9 Department of International Relations and Information Technology, University of Veterinary Science, Yezin, Yeyathiri, Nay Pyi Taw 15013, Myanmar; drhminoo@gmail.com; 10 Myanmar Apiculture Association, Yangon 11021, Myanmar; heinthuaung86@gmail.com; 11 Department of Food Science and Biotechnology, Gyeongkuk National University, 1375 Gyeongdong-Ro, Andong-Si 36729, Gyeongsangbuk-Do, Republic of Korea; jeehye@gknu.ac.kr

**Keywords:** mead, honey, aging, volatile organic compounds (VOCs), electronic nose

## Abstract

Mead quality is shaped by both fermentation and subsequent bottle aging, but stage-resolved descriptions that follow one production batch from honey must through fermented mead to aged mead remain scarce, particularly for region-specific single-honey products. Here, mead produced from *Tithonia diversifolia* honey from Southern Shan State, Myanmar, was characterised at three processing stages: pre-fermentation must (Pre), post-fermentation mead (Post), and mead bottle-aged for 1 year under ambient conditions at 24–28 °C (Aged). Physicochemical parameters were measured in three biologically independent production replicates and analysed statistically. Volatile and non-volatile profiling was performed on a single composite sample per stage, prepared by combining the three replicates, and is therefore descriptive: it characterises one production batch and does not support statistical comparison between stages. Fermentation reduced glucose and fructose from 71.9 ± 0.2 and 86.6 ± 0.8 mg/mL to below the limit of quantification, and both remained below it after aging. Mead pH rose slightly during fermentation (3.80 ± 0.07 to 3.88 ± 0.05) and fell during storage (3.67 ± 0.05), while colour shifted towards the lighter end of the Pfund scale rather than browning. Semi-quantitative electronic-nose analysis detected 60 putative volatile candidates across the sequence. The pre-fermentation profile was the least similar to the others (Pre versus Post, *r* = 0.16; Pre versus Aged, *r* = 0.04, against *r* = 0.38 between Post and Aged), and the post-fermentation profile was dominated by alcohol- and sulfur-associated responses. Twenty-nine candidates were detected only after fermentation, and 17 were shared between the fermented and aged mead, while the aged sample additionally contained candidates, including pentanol, 2-methylpropanoic acid, propyl acetate, 2-methylbutanal, thiophene, and terpinen-4-ol. Untargeted UHPLC-QTOF/MS resolved 10 non-volatile features that differed in detection across the three stages; these were putatively annotated by accurate mass, isotopic fidelity, and MS/MS library matching, with peptides and peptide derivatives forming the largest group. Because no authentic standards were analysed, these annotations remain tentative. Because the profiling data derive from one batch of one monofloral honey fermented with one yeast strain and were not confirmed by orthogonal or sensory methods, the compounds reported here define a candidate feature list for targeted validation in *T. diversifolia* mead rather than established markers of mead maturation.

## 1. Introduction

Mead is a honey-based alcoholic beverage produced by fermenting diluted honey with yeast. Its final composition is shaped by the starting honey matrix, fermentation conditions, yeast metabolism, and post-fermentation maturation [1,2,3,4]. Interest in mead has grown in parallel with the expansion of artisanal and premium fermented beverage markets, where product differentiation increasingly depends on aroma complexity, regional authenticity, and process control [4,5]. From an analytical perspective, mead is also a chemically distinctive system because honey provides a highly variable matrix of sugars, organic acids, amino acids, minerals, phenolics, terpenoid-related compounds, and other minor constituents that can influence both yeast metabolism and the volatile profile of the final beverage [1,3,5].

Current evidence indicates that mead aroma is determined largely by the balance among alcohols, esters, acids, aldehydes, terpenes, phenolic compounds, furans, and sulfur-containing volatiles, many of which are generated, transformed, or reweighted during fermentation and later maturation [1,3,6]. Fermentation is especially important because yeast metabolism does not merely convert sugars into ethanol and carbon dioxide but also produces a wide array of aroma-active secondary metabolites through central carbon metabolism and amino acid catabolism, including higher alcohols, acetate esters, ethyl esters, carbonyl compounds, and sulfur-related volatiles [6,7]. At the same time, the starting honey matrix remains important because botanical origin affects the initial volatile composition and precursor availability, thereby influencing both the direct inherited aroma background and the chemistry that unfolds during fermentation [2,8].

Among the factors influencing mead volatile composition, bottle aging remains comparatively less resolved at the level of stage-specific chemical change from must through fermentation to extended storage. In fermented beverages more broadly, aging can alter aroma through ester hydrolysis and re-esterification, oxidation and redox-driven reactions, acid rebalancing, and slower physicochemical interactions that reshape the relative contribution of pre-existing compounds rather than simply adding or removing single components [9,10,11]. Recent mead work has likewise shown that 1 year aging can substantially modify aroma profiles and reduce off flavour expression while preserving a clear dependence on the chemistry established during fermentation. However, compared with grape wine and some other fermented beverages, mead still lacks enough longitudinal datasets that follow the same batch from pre-fermentation must through post-fermentation product to extended aging. This limits the development of marker-based approaches for maturation monitoring, quality control, and product differentiation [4].

Botanical honey origin adds a further layer of complexity to this problem. Honey volatile composition is widely used as an indicator of botanical origin, and honey source may influence the sensory identity of the final mead through both direct transfer of aroma related compounds and indirect effects on fermentation chemistry [8,12]. In our previous work on meads produced from five Mekong region honeys, including tree marigold (*Tithonia diversifolia*) honey, fermentation caused systematic changes in physicochemical properties and biological activities, confirming that honey origin is not a trivial starting factor but an important biochemical determinant of mead characteristics [13]. However, that study focused on the transition from honey must to post-fermentation mead and did not examine longer term aging, which is highly relevant to real storage practice and premium product positioning.

To address this gap, the present study followed *T. diversifolia* honey mead across three defined processing stages: pre-fermentation must (Pre), post-fermentation mead (Post), and mead bottle-aged for 1 year (Aged). We combined biologically replicated physicochemical measurements with representative volatile and non-volatile compositional profiling to examine how fermentation and aging reshape the chemical properties of this mead system. Specifically, we aimed to determine which physicochemical changes accompany fermentation, which volatile chemical classes dominate the transition from must to fermented mead, and which stage-associated chemical features characterize 1-year bottle aging.

We hypothesized that fermentation would produce the dominant shift in the mead chemical profile through sugar depletion and expansion of yeast-associated alcohol, ester, and sulfur-containing signals, whereas bottle aging would further refine the post-fermentation matrix through selective changes in acids, aldehydes, esters, lactones, and other stage-associated volatile candidates. By resolving these transitions within one region-specific honey mead system, this study strengthens the chemical basis for quality development, maturation assessment, and future candidate validation in premium honey wines.

## 2. Materials and Methods

### 2.1. Mead Production and Experimental Design

*Tithonia diversifolia* honey was harvested in November 2023 from hives located within flowering *T. diversifolia* stands near Aungban, Kalaw Township, Southern Shan State, Myanmar (20.658042° N, 96.649668° E). The presence of *T. diversifolia* pollen was verified by microscopic examination and comparison with a reference pollen database at the SMART BEE SDGs Laboratory, Chiang Mai University. The honey was stored in the dark until use. Mead was prepared following the general procedure previously described for Mekong region honey meads by Inwongwan et al. [13], with adaptation for the present aging study. Honey must was prepared by diluting the honey with DI water to 25 °Brix, corresponding to an initial specific gravity of 1.100 measured with a calibrated hydrometer. Three independent glass vessels were each filled with 4 L of must, leaving a headspace of about 200 mL, and fitted with airlocks. Each vessel was inoculated with 1.0 g of commercial Lalvin EC-1118 *Saccharomyces cerevisiae* active dried wine yeast (Lallemand Oenology, Montreal, QC, Canada), corresponding to the manufacturer-recommended inoculation rate of 0.25 g/L (25 g/hL). The yeast was rehydrated according to the manufacturer’s package instructions before pitching. This inoculation rate was expected to provide an initial cell density of approximately 5 × 10^6^ viable cells/mL, based on the manufacturer’s specification; viable cell density was not independently determined. Fermentation proceeded in the dark at 25 ± 3 °C without agitation. Fermentation was judged to be complete after approximately 25 days, when visible carbon dioxide evolution had ceased and the mead had clarified progressively. Completion was therefore established from these visual criteria rather than from a numerical endpoint: specific gravity was recorded at the start of fermentation but not at its conclusion, and final gravity, ethanol concentration, and titratable acidity were not determined. This limitation is stated explicitly in Section 4.5.

After fermentation, the mead was racked off the lees and transferred into 750 mL clear glass wine bottles, leaving a headspace of approximately 25 mL. Bottles were closed with crown caps. No sulfite or other preservative was added, and neither dissolved nor headspace oxygen was measured. Bottles were stored upright in the dark at ambient room temperature, which was observed to range between 24 and 28 °C over the 12-month storage period. This storage condition was chosen deliberately. Climate-controlled cellaring is uncommon among artisanal producers in Northern Thailand and the wider Greater Mekong Subregion, where bottled products are typically held at ambient room temperature, so 24–28 °C represents the storage scenario that is relevant to this regional product rather than a controlled reference condition.

Samples for the analysis were collected at three defined processing stages: pre-fermentation must (Pre), post-fermentation mead (Post), and mead bottle-aged for 1 year (Aged). For core physicochemical analyses, including pH, colour, and residual sugar determination, the three biological replicates were retained and analyzed independently at each stage. Where technical replicate measurements were performed, technical values were first averaged within each biological replicate before statistical analysis. For broad chemical profiling, equal aliquots from the three biological replicates were combined at each stage to generate one homogeneous pooled-stage composite. These composites were used for electronic-nose and UHPLC-QTOF/MS analyses. Pooling was chosen to obtain a stable pooled-stage composite for discovery-level screening and to standardise sample preparation across stages. Within the resources available for a single profiling campaign, biological replication was allocated to the parameters on which statistical claims were intended, namely pH, colour, and residual sugars, each measured in three independent vessels, while composite sampling was used for the exploratory volatile and non-volatile profiling. We note explicitly that this choice removes biological replication from the electronic-nose and UHPLC-QTOF/MS datasets: each stage is represented by one measured profile, and the subsequent injections are analytical replicates that quantify instrument repeatability rather than variation between fermentations. All conclusions drawn from these two datasets are correspondingly descriptive and apply to this production batch.

Pre samples were collected on 19 May, 2024, Post samples on 14 June, 2024, and Aged samples on 15 June, 2025, the last of these marking the completion of 12 months of bottle aging. All aliquots were transferred into sealed 50 mL tubes and held at −80 °C until analysis; instrumental acquisition and subsequent data processing were carried out between June 2025 and May 2026. Because sampling necessarily followed the production timeline, the Pre and Post aliquots had been held in frozen storage for approximately 13 and 12 months, respectively, by the time the Aged samples were collected, whereas the Aged aliquot entered storage immediately before analysis. Differential frozen-storage duration is therefore confounded with processing stage, as noted in Section 4.5. All three composite samples were prepared on the same day and analysed within a single continuous instrumental sequence using the same column, mobile phases, calibration procedure, and acquisition settings, with injection order randomised to minimise run-order effects. No pooled quality-control sample was injected. Analysing the composites together minimised inter-run analytical variation but did not correct the unequal storage periods; sampling point and frozen-storage history are therefore inseparable in this design, and the contribution of frozen storage can be neither quantified nor excluded.

### 2.2. Colour Measurement and pH of Honey Mead

Colour and pH were measured independently for each of the three biological production replicates at each processing stage. Where repeated technical measurements were obtained, these readings were averaged to provide one value for each biological replicate. Colour was measured using a HI 96785 Honey Colour Portable Photometer (Hanna Instruments, Woonsocket, RI, USA). The instrument was calibrated according to the manufacturer’s instructions before analysis. Each sample was gently mixed, transferred into a clean cuvette without visible bubbles or suspended particles, and measured in honey colour mode. Results were expressed on the Pfund scale and assigned to the corresponding honey colour classification. pH was measured using a calibrated pH meter following the general AOAC procedure for honey-based samples. Briefly, 4 g of the sample were diluted with 30 mL of distilled water and mixed until homogeneous. The pH meter was calibrated with standard buffer solutions at pH 4.00, 7.00, and 10.00 before analysis. The electrode was immersed in the prepared solution, and the pH was recorded after stabilization [13]. The electrode was rinsed with distilled water between measurements. Results are reported as mean ± standard deviation from three biologically independent production replicates.

### 2.3. Sugar Analysis Using HPLC

Samples were diluted as required with deionized water and filtered through 0.45 µm nylon membrane syringe filters before HPLC injection. Pre samples were diluted 10-fold to bring glucose and fructose concentrations within the 1–10 mg/mL calibration range, and all dilution factors were incorporated into the final concentration calculations. Residual sugars in the mead samples were quantified by high-performance liquid chromatography using an Agilent 1260 system equipped with a diode array detector and refractive index detector (Agilent Technologies, Santa Clara, CA, USA). Glucose and fructose were separated on an Asahipak NH2P 50 4EE column (4.6 mm internal diameter × 250 mm length; Shodex, Resonac Corporation, Tokyo, Japan), which is suitable for carbohydrate analysis. The analysis was performed under isocratic conditions using acetonitrile and deionized water at a ratio of 75:25 (*v*/*v*) as the mobile phase. The flow rate was set at 1.0 mL/min, the column temperature was maintained at 30 °C, and the injection volume was 10 µL. Each chromatographic run was continued for 120 min to allow sufficient separation of the target sugars [13]. For quantification, external calibration curves were prepared using glucose and fructose standards at six concentration levels: 1, 2, 4, 6, 8, and 10 mg/mL. Each standard concentration was analysed in triplicate. Sugar concentrations in the samples were calculated from the corresponding calibration curves and expressed as mg/mL. All sample analyses were conducted in triplicate, and results were presented as mean ± standard deviation.

### 2.4. UHPLC-QTOF/MS Analysis and Compound Annotation

Phytochemical characterization of the mead samples was carried out using a UHPLC-QTOF/MS platform (Bruker Daltonics GmbH & Co. KG, Bremen, Germany) coupled to a Bruker Impact II QTOF mass spectrometer and fitted with a Bruker Intensity Solo 1.8 C18-2 analytical column (100 × 2.1 mm, 1.8 μm particle size). The column temperature was maintained at 35 °C throughout the analysis. For each processing stage, samples previously stored at −80 °C in tightly sealed 50 mL tubes were thawed at 4 °C. For each pooled stage composite, 45 mL of sample were transferred into a 50 mL tube and centrifuged at 5000 rpm for 5 min at 4 °C to remove suspended cells and insoluble material. The mead samples were diluted with an equal volume of acetonitrile and water (1:1, *v*/*v*), followed by vortex mixing for approximately 5 min to ensure homogeneity, and the solution was clarified through a 0.45 μm nylon membrane filter. This acetonitrile dilution was specific to UHPLC-QTOF/MS sample preparation and was not used for HPLC sugar quantification. Because the UHPLC-QTOF/MS results were interpreted as qualitative presence-and-absence annotations rather than compound concentrations, no concentration correction was applied. Separation of phytochemicals was achieved using a binary solvent system consisting of 0.1% formic acid in water (mobile phase A) and 0.1% formic acid in methanol (mobile phase B). The chromatographic gradient was programmed from 1% B at 0–10 min, increased to 99% B from 10–15 min, and returned to 1% B from 15–20 min. The flow rate was fixed at 0.2 mL/min, and 2 μL of each sample were introduced into the system [14].

Mass spectrometric detection was conducted under electrospray ionization (ESI) conditions in both positive and negative acquisition modes. Instrumental settings included a capillary voltage of 3.6 kV, nebulizer gas pressure of 2.2 bar, and a drying gas flow of 10.0 L/min maintained at 220 °C. Accurate-mass spectra were collected over an *m*/*z* range of 100–1000 with an acquisition rate of three spectra per second.

The resulting datasets were processed in Bruker Compass DataAnalysis (version 6.2) for untargeted screening. Features were extracted and molecular formulae assigned from accurate mass, isotopic fidelity, and MS/MS fragmentation acquired in both ionization modes. Theoretical *m*/*z* values were calculated from IUPAC monoisotopic atomic masses for the ion actually detected, using the proton mass (1.007276 Da) rather than the hydrogen atom mass; sodiated species are reported as [M+Na]^+^ against the corresponding theoretical value and deprotonated species as [M−H]−. Mass error was calculated as 10^6^ × (*m*/*z* measured − *m*/*z* theoretical)/*m*/*z* theoretical and is reported in ppm. Annotations were retained only when the mass error was ≤10 ppm, the isotopic pattern was consistent with the proposed molecular formula, and the MS/MS library match score was ≥700; features failing any criterion were discarded. Where more than one molecular formula or isomeric structure was consistent with a measured mass, the highest-ranked candidate was reported, and the feature was counted once. Because no authentic standards were analysed, assignments correspond at best to level 2 (probable structure, supported by library MS/MS match) or level 3 (tentative candidate) of the Schymanski scale, and they are reported throughout as putative annotations rather than confirmed identifications. No pooled quality-control sample was included in the analytical sequence; instrument stability was instead monitored from the replicate injections of each composite sample acquired within the same sequence.

### 2.5. Electronic-Nose Analysis

Volatile organic compounds present in the mead samples were characterized using a Heracles II ultra-fast gas chromatography electronic-nose system (Alpha M.O.S., Toulouse, France) fitted with an MXT-5 capillary column (10 m × 0.18 mm × 0.40 µm) and flame ionization detection. The samples were equilibrated at 60 °C for 5 min prior to analysis. A 5 mL portion of the resulting headspace was then introduced into the instrument at an injection rate of 125 µL/s. During analysis, the injector and detector temperatures were maintained at 200 °C and 260 °C, respectively, whereas the trapping system was operated at 40 °C with a split flow of 10 mL/min. The chromatographic run commenced at 50 °C, followed by a gradual increase to 80 °C at 1 °C/s, a subsequent increase to 250 °C at 3 °C/s, and the final temperature was maintained for 21 s [14]. All determinations were conducted in triplicate. Putative compound identification was done through comparison with reference spectra contained in the AroChemBase database using the instrument’s sensory analysis software. Only VOC candidates with database match scores of ≥80% were retained for exploratory interpretation.

These assignments are database matches based on retention behaviour and were not verified against authentic standards or by GC-MS; they correspond to tentative candidate identifications (Schymanski level 3) and are referred to throughout as putative VOC candidates, with compounds detected in only one stage described as stage-associated candidates rather than as markers of that stage. Semi-quantitative abundance was estimated from normalised chromatographic peak areas against an external hexanal calibration curve prepared over the range 0.001–100 µL/mL, with each level injected in triplicate, and it is expressed throughout as µg/L hexanal equivalents. Because a single-analyte calibrant was used, compound-specific recovery, limits of detection and quantification, and precision were not determined for the individual analytes; the reported values are hexanal-equivalent detector responses intended for comparison between processing stages rather than absolute concentrations. Flame-ionisation response factors and headspace partition coefficients differ substantially among alcohols, esters, aldehydes, carboxylic acids, and sulfur-containing compounds, so the same detector response corresponds to different true concentrations for different analytes. For the same reason, the sums of responses within a chemical class indicate the relative magnitude of a class-level signal and must not be read as the total concentration of that class. Responses falling below the lowest calibration level lie outside the calibrated range and are reported only as trace-level detections for presence-and-absence purposes.

### 2.6. Data Processing and Statistical Analysis

Inferential statistics were applied only to the physicochemical dataset (pH, colour, glucose, and fructose), which was obtained from three biologically independent production replicates at each processing stage. Where technical replicate measurements were performed, values were averaged within each biological replicate before analysis. For each parameter, a linear mixed-effects model was fitted with processing stage as a fixed effect and biological replicate identity as a random effect, and pairwise comparisons were adjusted using Tukey’s method, with significance set at *p* < 0.05.

No inferential statistics were applied to the electronic-nose or UHPLC-QTOF/MS data. For these analyses, equal aliquots of the three biological replicates were combined into one composite sample per stage, so each stage is represented by a single measured profile, and the replicate injections performed on that composite are analytical rather than biological replicates. Variability among fermentation vessels therefore cannot be estimated from these data, no measure of dispersion can be attached to any stage, and no difference between stages can be tested for significance. These data are presented as a descriptive, single-batch compositional comparison. Principal component analysis was not applied to the three composite profiles because, with three mean-centred observations, the data occupy a two-dimensional subspace, and the first two components necessarily account for all of the variance, so any apparent separation would be a consequence of the number of samples rather than evidence of a difference between stages. Pairwise Pearson correlations between composite profiles and the abundance heatmap are presented as descriptive summaries of profile similarity. Analytical replicate injections were used only to assess measurement repeatability. All calculations were performed in R version 4.6.0.

## 3. Results

### 3.1. Stage-Dependent Physicochemical Changes and Stage-Resolved Volatile Chemical-Class Profiles

Physicochemical analysis showed clear stage-dependent changes in pH and residual sugars (Table 1). The pH differed among processing stages. Post-fermentation mead showed the highest pH value, 3.88 ± 0.05 and was higher than the 1-year aged mead, 3.67 ± 0.05, whereas the pre-fermentation must, 3.80 ± 0.07, was not different from either Post or Aged samples. Residual sugars showed the strongest physicochemical transition during fermentation. Glucose decreased from 71.90 ± 0.20 mg/mL in Pre to below the validated quantification range in both Post and Aged samples. Fructose similarly decreased from 86.60 ± 0.80 mg/mL in Pre to below the validated quantification range in Post and Aged samples. For both sugars, Pre was higher than the two later stages. These findings indicate that the major depletion of glucose and fructose occurred during fermentation, with only trace residual sugars detected after fermentation and bottle aging.

Exploratory UHPLC-QTOF/MS screening detected 10 putatively annotated non-volatile features that satisfied the ≤10 ppm mass-accuracy criterion of Section 2.4 (Table 2). Peptides and peptide derivatives formed the predominant class, accounting for five features (50%): a pentapeptide (Ala-Asn-Cys-Ser-Met, #4, RT 7.10 min, *m*/*z* 525.1801), a dipeptide (Ala-Lys, #5, RT 7.90 min, *m*/*z* 218.1498), a heptapeptide (Ser-Ala-Ser-Thr-Arg-Ala-Thr, #8, RT 12.40 min, *m*/*z* 693.3525), a tetrapeptide (Pro-Asn-Ser-Arg, #9, RT 12.50 min, *m*/*z* 473.2459), and a tetrapeptide derivative (Z-Val-Pro-Val-Pro-OH, #10, RT 13.10 min, *m*/*z* 545.2969). Heterocyclic compounds accounted for two features (20%), namely 1,2,4-thiadiazolidine-3,5-dione (#2, RT 3.70 min, *m*/*z* 116.9763) and 2,2-bis(2H-tetrazol-5-yl)dodecane-5,8-dione (#7, RT 11.60 min, *m*/*z* 357.1783). The remaining three features (10% each) comprised an aromatic heterocycle (2-methylthiophenothiazine, #1, RT 2.20 min, *m*/*z* 244.0267), a polyketide-derived quinone (tetrangulol, #3, RT 5.70 min, *m*/*z* 305.0821), and a sulfated carbohydrate derivative (1,3,5-tri-O-acetyl-4-O-methyl-fucitol-2-sulfate, #6, RT 10.30 min, *m*/*z* 409.0775). All 10 assignments are putative: they were obtained by accurate-mass, adduct, isotope-pattern, and MS/MS spectral matching without authentic standards, and for the peptide-related features, the elemental composition does not by itself establish the residue sequence.

Across processing stages, three of these features were detected in the pre-fermentation must, six in the post-fermentation mead, and six in the aged mead. Only one feature was detected at all three stages, whereas five features absent from the must were detected after fermentation, four of the six features detected after fermentation were still detected after 1 year of storage, and two features were detected only in the aged mead against two detected only in the must. The non-volatile fraction therefore follows the same broad pattern as the volatile data, in which the larger compositional change accompanied fermentation and aging was associated with a smaller, more selective difference. These are detection counts in a single composite sample per stage: they are not abundance measurements, they are not biologically replicated, and no interpretation offered here depends on the identity of any individual feature.

**Table 2:** Putatively annotated non-volatile features in the pooled stage composites of *T. diversifolia* honey mead.

#	RT (min)	Compound Name	Molecular Formula and Detected Ion	Mass (*m*/*z*)		Error (ppm)	Sample			Class
				Theoretical	Observed		Pre	Post	Aged	
1.	2.20	2-Methylthiophenothiazine	C_13_H_11_NS_2_ [M−H]^−^	244.026	244.0267	2.81	−	+	−	Aromatic heterocycle
2.	3.70	1,2,4-Thiadiazolidine-3,5-dione	C_2_H_2_N_2_O_2_S [M−H]^−^	116.9764	116.9763	−1.04	−	+	+	Heterocyclic organic compound
3.	5.70	Tetrangulol	C_19_H_12_O_4_ [M+H]^+^	305.0808	305.0821	4.15	+	−	−	Polyketide-derived quinone
4.	7.10	Ala-Asn-Cys-Ser-Met	C_18_H_32_N_6_O_8_S_2_ [M+H]^+^	525.1796	525.1801	0.99	+	−	−	Pentapeptide
5.	7.90	Ala-Lys	C_9_H_19_N_3_O_3_ [M+H]^+^	218.1499	218.1498	−0.54	−	+	+	Dipeptide
6.	10.30	1,3,5-Tri-O-acetyl-4-O-methyl-fucitol-2-sulfate	C_13_H_22_O_11_S [M+Na]^+^	409.0775	409.0775	−0.01	−	−	+	Sulfated carbohydrate derivative
7.	11.60	2,2-Bis(2H-tetrazol-5-yl)dodecane-5,8-dione	C_14_H_22_N_8_O_2_ [M+Na]^+^	357.1758	357.1783	7.02	−	+	+	Organoheterocyclic compound
8.	12.40	Ser-Ala-Ser-Thr-Arg-Ala-Thr	C_26_H_48_N_10_O_12_ [M+H]^+^	693.3526	693.3525	−0.13	−	−	+	Heptapeptide
9.	12.50	Pro-Asn-Ser-Arg	C_18_H_32_N_8_O_7_ [M+H]^+^	473.2467	473.2459	−1.63	+	+	+	Tetrapeptide
10.	13.10	Z-Val-Pro-Val-Pro-OH	C_28_H_40_N_4_O_7_ [M+H]^+^	545.297	545.2969	−0.14	−	+	−	Tetrapeptide derivative

Remarks: Data are from one pooled stage composite per sampling point. Retention time and measured *m*/*z* are direct instrument outputs. Theoretical *m*/*z* was calculated from IUPAC monoisotopic atomic masses for the detected ion given in the formula column, using the appropriate ionic/adduct mass; mass error (ppm) = 10^6^ × (measured − theoretical)/theoretical, and only features within ≤10 ppm were retained. All compound assignments are putative: they were obtained by accurate-mass, adduct, isotope-pattern, and MS/MS spectral matching, and none were confirmed with an authentic standard. Where more than one molecular formula or isomeric structure was consistent with a measured mass, the highest-ranked candidate is shown and the feature is counted once; for the peptide-related features, the elemental composition does not establish the residue sequence because all re-orderings of the same residues have identical mass. RT, retention time; +, detected under the applied analytical threshold; −, not detected under the applied analytical threshold.

**Figure 1:**
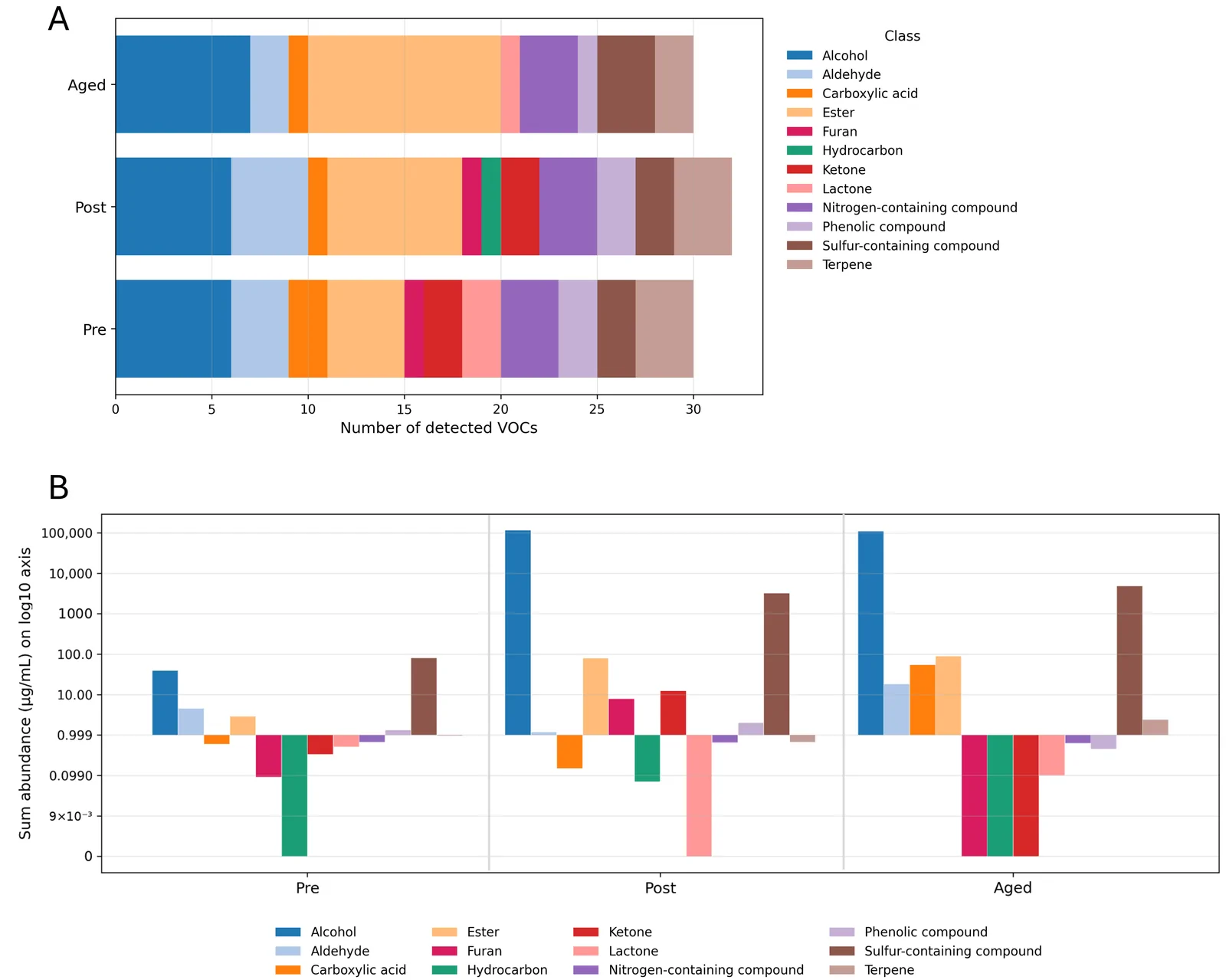
Stage-resolved distribution of putative VOC chemical classes in representative composite samples. (**A**) Number of putative VOC candidate detected in each chemical class across Pre, Post, and Aged stages. (**B**) Summed semi-quantitative abundance of putative VOC candidates within each chemical class, expressed as µg/L hexanal equivalents. Class-level sums combine compounds with different flame-ionisation response factors and headspace partition coefficients and are therefore comparative signal magnitudes, not concentrations. All compound names shown are putative electronic-nose database matches that were not confirmed with authentic standards or by GC-MS.

In addition to changes in the non-volatile phytochemical composition, substantial variations were observed in the volatile fraction responsible for aroma development during mead production and aging. Across all stages, 60 putative VOC candidates were detected in total, while within stage richness, they remained broadly similar, with 30 compounds in Pre, 32 in Post, and 30 in Aged. However, chemical class composition revealed clear stage-dependent differences. By compound count, ester diversity increased progressively from four compounds in Pre to seven in Post and ten in Aged, indicating a larger number of candidates in this class in the later stages. Alcohols remained consistently represented across stages, with six compounds detected in both Pre and Post and seven in Aged, whereas several minor classes showed stage specificity or nonmonotonic behaviour, including hydrocarbons that appeared only after fermentation and lactones that were detected in Pre, absent in Post, and reappeared in Aged (Figure 1A).

By summed abundance, the largest quantitative difference in the detected volatile pool was between Pre and Post (Figure 1B). Total alcohol abundance increased sharply from 38.76 µg/L in Pre to 1.15 × 10^5^ µg/L hexanal equivalents in Post and remained high in Aged at 1.09 × 10^5^ µg/L hexanal equivalents, indicating a strong fermentation associated expansion of the alcohol fraction that persisted during storage. Sulfur-containing compounds also increased markedly, from 79.28 µg/L hexanal equivalents in Pre to 3.19 × 10^3^ µg/L hexanal equivalents in Post and 4.83 × 10^3^ µg/L hexanal equivalents in Aged. Esters increased in absolute terms from 2.88 µg/L hexanal equivalents in Pre to 79.09 µg/L hexanal equivalents in Post and 88.20 µg/L hexanal equivalents in Aged but remained quantitatively minor relative to alcohols. In contrast, aging was distinguished by selective accumulation of carboxylic acids, which rose from 0.15 µg/L hexanal equivalents in Post to 53.48 µg/L hexanal equivalents in Aged, largely due to the appearance of 2-methylpropanoic acid. Together, these class level data indicate that the main volatile framework was already present after fermentation, whereas the aged sample differed more selectively.

### 3.2. Descriptive Comparison of the Three Pooled VOC Composites

**Figure 2:**
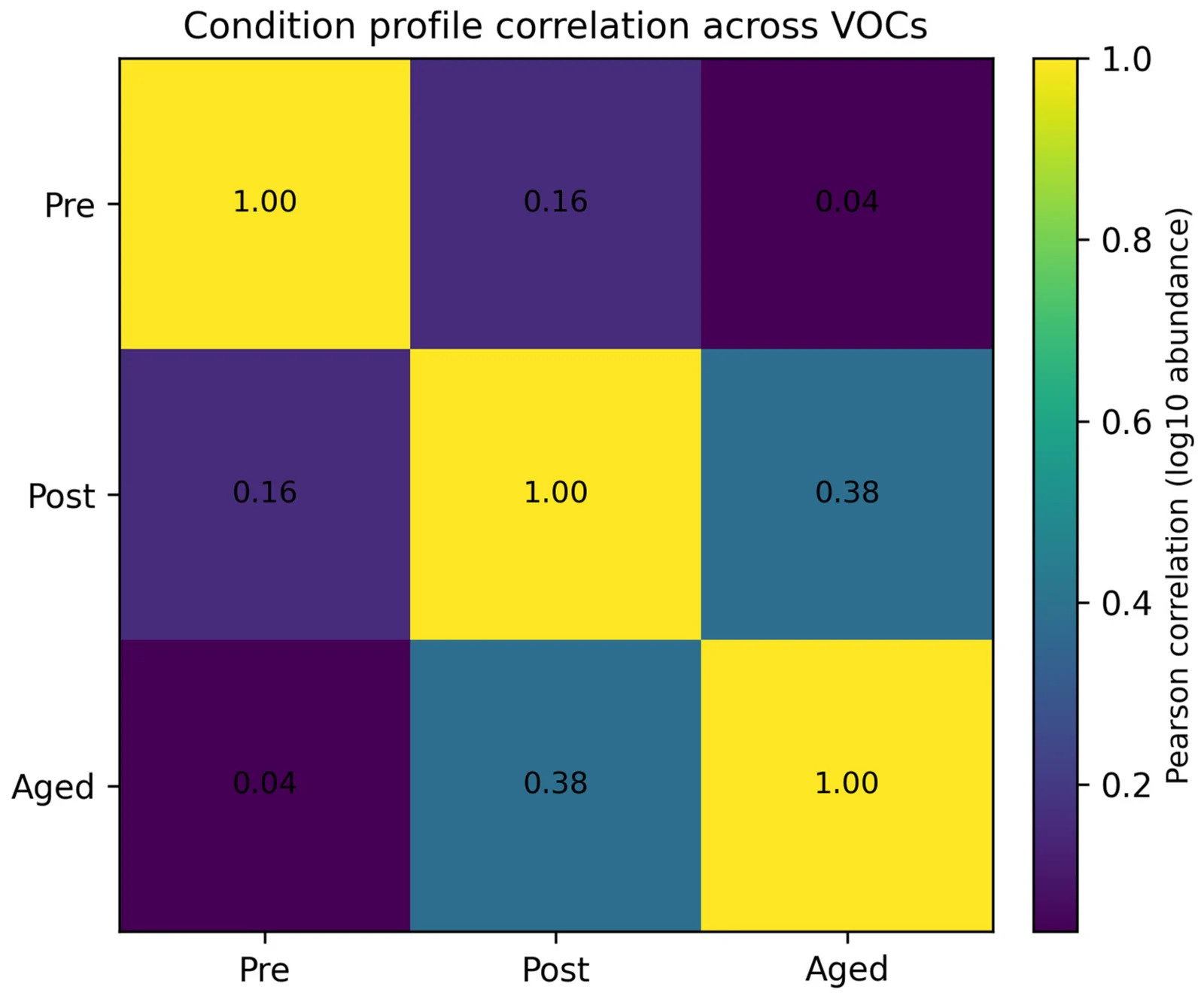
Pearson correlation between the volatile profiles of the three pooled stage composites. Each coefficient is a single pairwise comparison between two composite profiles and is descriptive only; no statistical inference about differences between stages can be drawn from three composite samples. Pre, pre-fermentation must; Post, post-fermentation mead; Aged, mead bottle-aged for 1 year.

Pairwise Pearson correlations were calculated between the three pooled VOC composites (Figure 2). The pre-fermentation profile showed low similarity to both later stages (Pre versus Post, *r* = 0.16; Pre versus Aged, *r* = 0.04), whereas the post-fermentation and aged profiles were more similar to each other (*r* = 0.38). Each coefficient is a single pairwise comparison between two composite profiles: it describes how alike the two measured profiles are and is not an estimate of a population correlation, so no confidence interval or significance can be attached to it. Interpreted at that level, the pattern is consistent with the compound-level results below, in which the aged sample retained a large part of the post-fermentation composition while both differed substantially from the pre-fermentation must. Compounds detected in only one stage are described in Section 3.4 and are referred to throughout as stage-associated candidates; because they derive from one composite sample per stage, they cannot be distinguished from compounds that happen to differ between individual fermentation vessels.

### 3.3. Hierarchical Clustering of VOC Abundance Patterns Indicates Fermentation-Anchored Modules and an Aging-Responsive Subset

**Figure 3:**
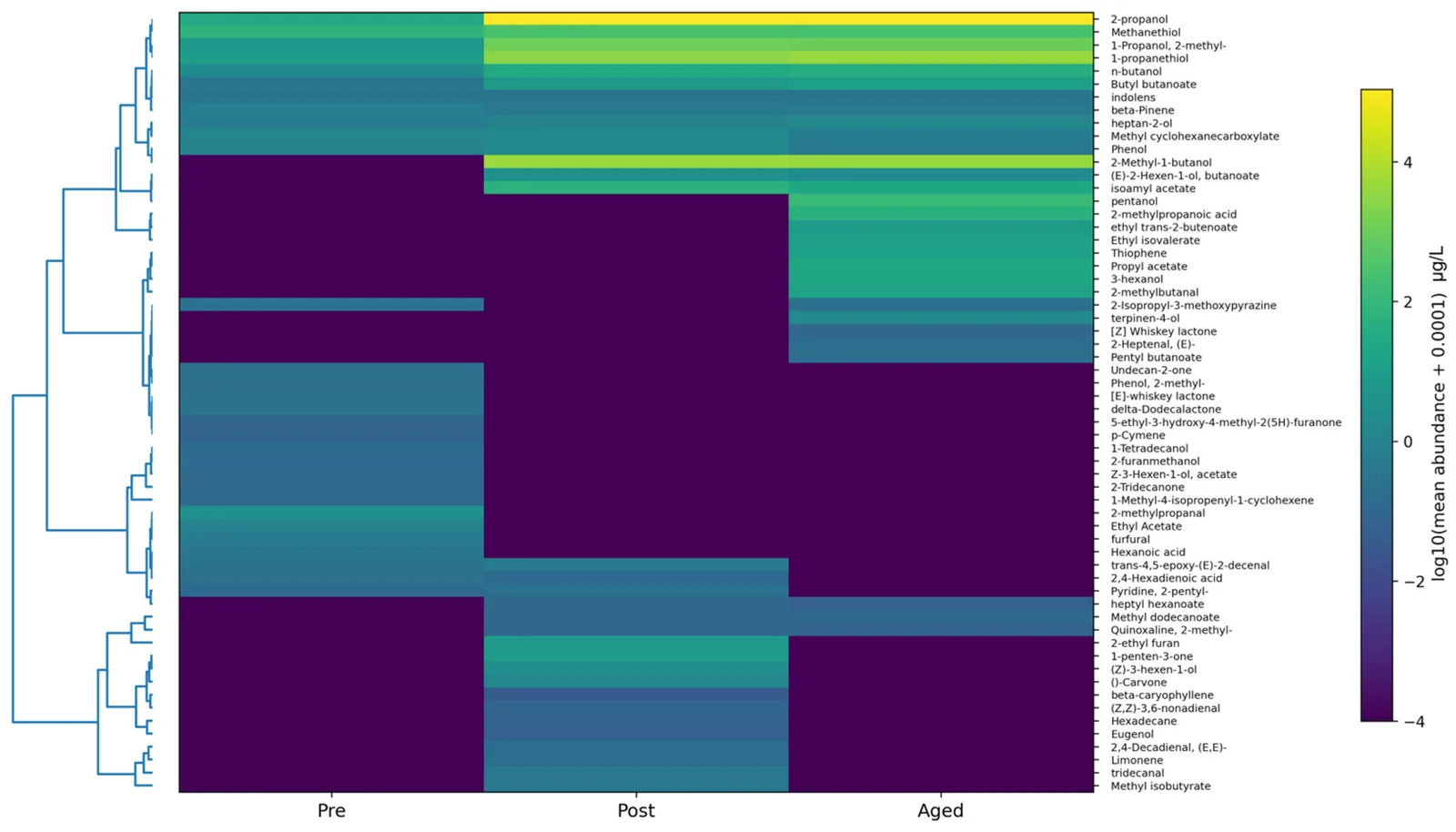
Hierarchical clustering of VOC abundance patterns across processing stages. Heatmap of scaled VOC abundances in Pre, Post, and Aged samples. Rows represent compounds and columns represent stages. Row groupings indicate Pre-enriched compounds, compounds detected only after fermentation and retained after aging, and compounds detected at higher response in Aged. Columns are single composite samples rather than replicated groups; no column dendrogram is shown, and no clustering support can be calculated from three composite profiles. Pre, pre-fermentation honey must; Post, mead after fermentation; Aged, mead after 12 months of bottle aging.

Hierarchical clustering of mean VOC abundances resolved coordinated stage-dependent modules of compounds and reinforced the dominant transition from Pre to the two later stages (Figure 3). The largest module comprised compounds that were absent or low in Pre, increased strongly after fermentation, and remained relatively elevated in Aged, indicating that the 1-year aged profile was built upon a fermentation-established backbone rather than a newly reconstructed composition. Representative compounds in this group included major alcohol- and sulfur-associated features such as 2-propanol, 2-methyl-1-butanol, and 1-propanethiol. A second subset showed stronger enrichment in Aged than in Post, indicating selective secondary modulation during storage. This group included candidates detected only in the aged sample, such as 2-methylpropanoic acid, pentanol, propyl acetate, 2-methylbutanal, thiophene, and terpinen-4-ol, consistent with a targeted maturation-related shift rather than uniform persistence of all fermentation-derived compounds.

In contrast, several compounds were enriched in Pre and declined or disappeared after fermentation, including 1-tetradecanol, 2-furanmethanol, furfural, hexanoic acid, [E]-whiskey lactone, and delta-dodecalactone. These compounds likely represent the pre-fermentation honey-derived baseline, which was substantially down-weighted in the profiles measured after fermentation. Taken together, the clustering pattern supports a two-step model in which fermentation establishes the main chemical scaffold, whereas aging selectively reshapes a smaller subset of features. The same ordering was apparent in the pairwise correlations (Figure 2), in which the pre-fermentation must was the least similar profile while the post-fermentation and aged samples were more alike.

### 3.4. Persistent and Stage-Associated VOC Candidates Across Mead Production

**Figure 4:**
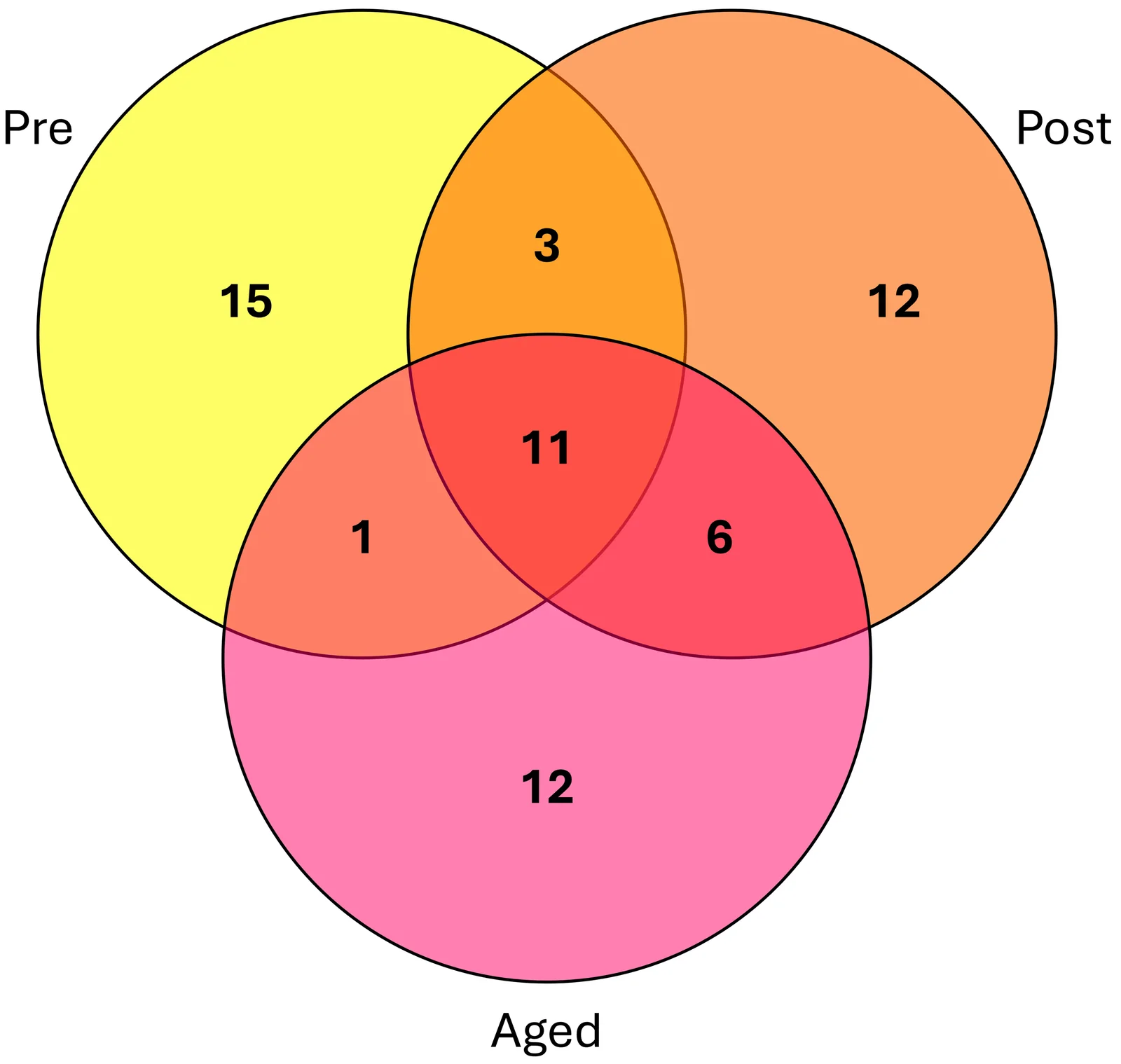
Shared and stage-associated putative VOC candidates across the mead-production sequence. The Venn diagram summarizes VOC candidates detected under the applied analytical threshold in representative Pre, Post, and Aged composite samples. Numbers indicate candidate counts within each intersection. All compound names shown are putative electronic-nose database matches that were not confirmed with authentic standards or by GC-MS.

Presence- and absence-based intersection analysis revealed substantial VOC identity turnover across processing stages while also identifying a stable core set retained throughout the full sequence from must to aged mead (Figure 4). Only candidates with reportable semi-quantitative values in both compared stages were included in the fold-change calculations; trace-level signals were excluded. Of the 60 compounds detected in total, 11 were shared across Pre, Post, and Aged, indicating that part of the volatilome persisted throughout fermentation and 1 year of storage. However, each stage also contained a substantial unique fraction, with 15 compounds detected only in Pre, 12 only in Post, and 12 only in Aged. This pattern shows that process progression altered compound identity, as well as abundance. Restricted overlaps between stages were unevenly distributed. Only three compounds were shared exclusively between Pre and Post, and only one was shared exclusively between Pre and Aged, whereas six compounds were shared exclusively between Post and Aged. The greater overlap between Post and Aged than between Pre and the later stages is consistent with the multivariate analysis, indicating that the aged product retained a substantial component of the post-fermentation chemical framework while acquiring additional stage-associated features. Candidates detected exclusively in one stage are described here as stage-associated candidates rather than definitive process markers. Because the mead was sampled only at the end of fermentation and after 12 months of storage, these observations describe the difference between two endpoints. They do not resolve when during storage any change occurred, and a compound detected only in the aged sample may have appeared early in storage and persisted or have appeared late; no maturation kinetics can be inferred from this design. Together, these descriptive abundance patterns are consistent with a two-stage profile in which fermentation produced the principal shift among persistent VOC candidates, whereas bottle aging was associated with more selective modulation of ester-, phenolic-, sulfur-, and alcohol-related features.

### 3.5. Stage-Associated Putative VOC Candidates and Database-Derived Descriptor Context

**Figure 5:**
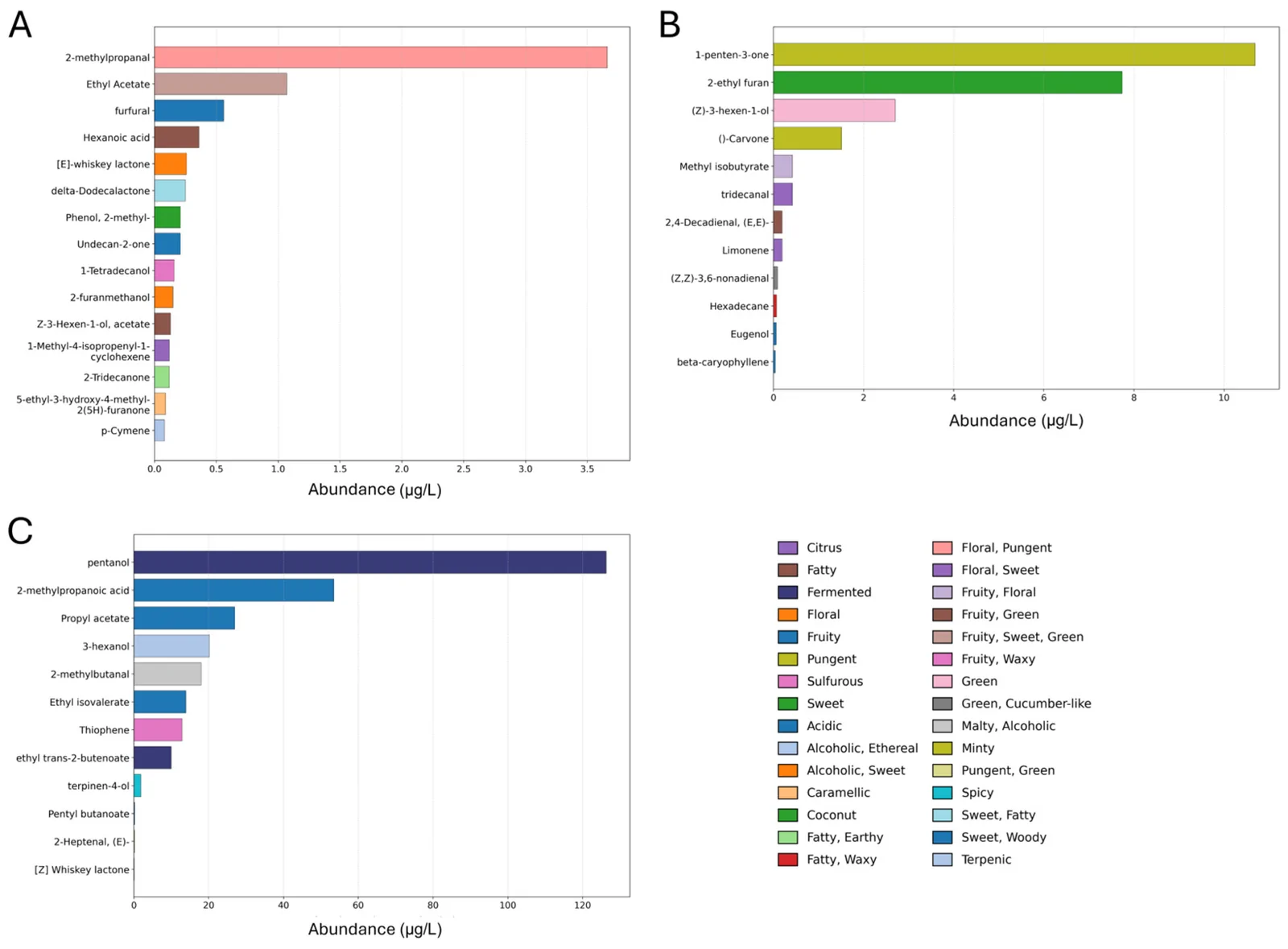
Ranked stage-specific VOC candidates and primary descriptor categories. Candidates detected only in (**A**) Pre, (**B**) Post, and (**C**) Aged samples, ranked by mean response within each stage and coloured by the primary descriptor category assigned from the first listed descriptor in the dataset. All compound names shown are putative electronic-nose database matches that were not confirmed with authentic standards or by GC-MS. Pre, pre-fermentation honey must; Post, mead after fermentation; Aged, mead after 12 months of bottle aging.

To derive interpretable stage discriminant compounds from the intersection analysis (Figure 4), VOCs unique to each processing stage were ranked by mean abundance within that stage and annotated using the primary sensory category derived from the first listed descriptor in the dataset (Figure 5). This approach highlights stage-specific candidate profiles while retaining a descriptor-based link to potential sensory directionality.

Pre-fermentation unique VOCs were collectively low in abundance and chemically diverse, consistent with a honey must baseline preceding the volatile expansion observed after fermentation. The most abundant Pre-specific compounds were 2-methylpropanal (3.66 µg/L hexanal equivalents), ethyl acetate (1.07 µg/L hexanal equivalents), and furfural (0.56 µg/L hexanal equivalents), followed by hexanoic acid (0.36 µg/L hexanal equivalents), trans-4,5-epoxy-(E)-2-decenal (0.27 µg/L hexanal equivalents), [E]-whiskey lactone (0.26 µg/L hexanal equivalents), delta-dodecalactone (0.25 µg/L hexanal equivalents), 2-isopropyl-3-methoxypyrazine (0.24 µg/L hexanal equivalents), and 5-ethyl-3-hydroxy-4-methyl-2(5H)-furanone (0.09 µg/L hexanal equivalents). Descriptor coding of this unique set suggested a comparatively diffuse baseline associated mainly with floral, fruity, sweet, fatty, waxy, earthy, and woody linked directions, rather than domination by a small number of highly abundant compounds (Figure 5A).

Post-fermentation exhibited a distinct stage-specific candidate profile dominated by fermentation associated ketone, furan, alcohol, and terpene-type compounds. The Post-specific candidates with the highest responses were 1-penten-3-one (10.69 µg/L hexanal equivalents), 2-ethyl furan (7.74 µg/L hexanal equivalents), and (Z)-3-hexen-1-ol (2.70 µg/L hexanal equivalents), followed by (−)-carvone (1.51 µg/L hexanal equivalents), tridecanal (0.42 µg/L hexanal equivalents), methyl isobutyrate (0.42 µg/L hexanal equivalents), pyridine, 2-pentyl- (0.24 µg/L hexanal equivalents), limonene (0.19 µg/L hexanal equivalents), 2,4-decadienal, (E,E)- (0.19 µg/L hexanal equivalents), quinoxaline, 2-methyl- (0.10 µg/L hexanal equivalents), hexadecane (0.07 µg/L hexanal equivalents), and eugenol (0.06 µg/L hexanal equivalents). Primary descriptor coding indicated stronger contributions from pungent, sweet, green, minty, floral, fatty, citrus, roasted, and woody-associated directions, consistent with the emergence of process-generated compounds after active fermentation (Figure 5B).

In contrast, the 1-year aged unique set was strongly skewed toward a smaller number of high-abundance compounds, indicating that specific VOCs were detected at responses that dominated the set of candidates detected only in Aged. Pentanol (126.31 µg/L hexanal equivalents) and 2-methylpropanoic acid (53.48 µg/L hexanal equivalents) were the dominant Aged-specific compounds, followed by propyl acetate (26.97 µg/L hexanal equivalents), 3-hexanol (20.24 µg/L hexanal equivalents), 2-methylbutanal (17.98 µg/L hexanal equivalents), ethyl isovalerate (13.90 µg/L hexanal equivalents), thiophene (12.85 µg/L hexanal equivalents), ethyl trans-2-butenoate (9.98 µg/L hexanal equivalents), terpinen-4-ol (1.85 µg/L hexanal equivalents), pentyl butanoate (0.21 µg/L hexanal equivalents), 2-heptenal, (E)- (0.17 µg/L hexanal equivalents), and [Z]-whiskey lactone (0.10 µg/L hexanal equivalents). Descriptor coding suggested that this Aged-specific set extended mainly toward fermented or alcoholic, acidic, fruity, sulfurous, and malty-associated directions, with additional coconut, pungent, green, and spicy linked features (Figure 5C). Overall, the ranked stage-specific candidate profiles are consistent with a progression from a chemically diverse but low-abundance Pre baseline, to a Post profile enriched in ketone- and furan-related candidates detected after fermentation, to an Aged profile dominated by a smaller number of high abundance alcohol and acid-related compounds with additional ester and sulfur contributions.

### 3.6. Pairwise Modulation Within Persistent VOC Pools Reveals Dominant Fermentation Effects and Selective Aging Fine-Tuning

**Figure 6:**
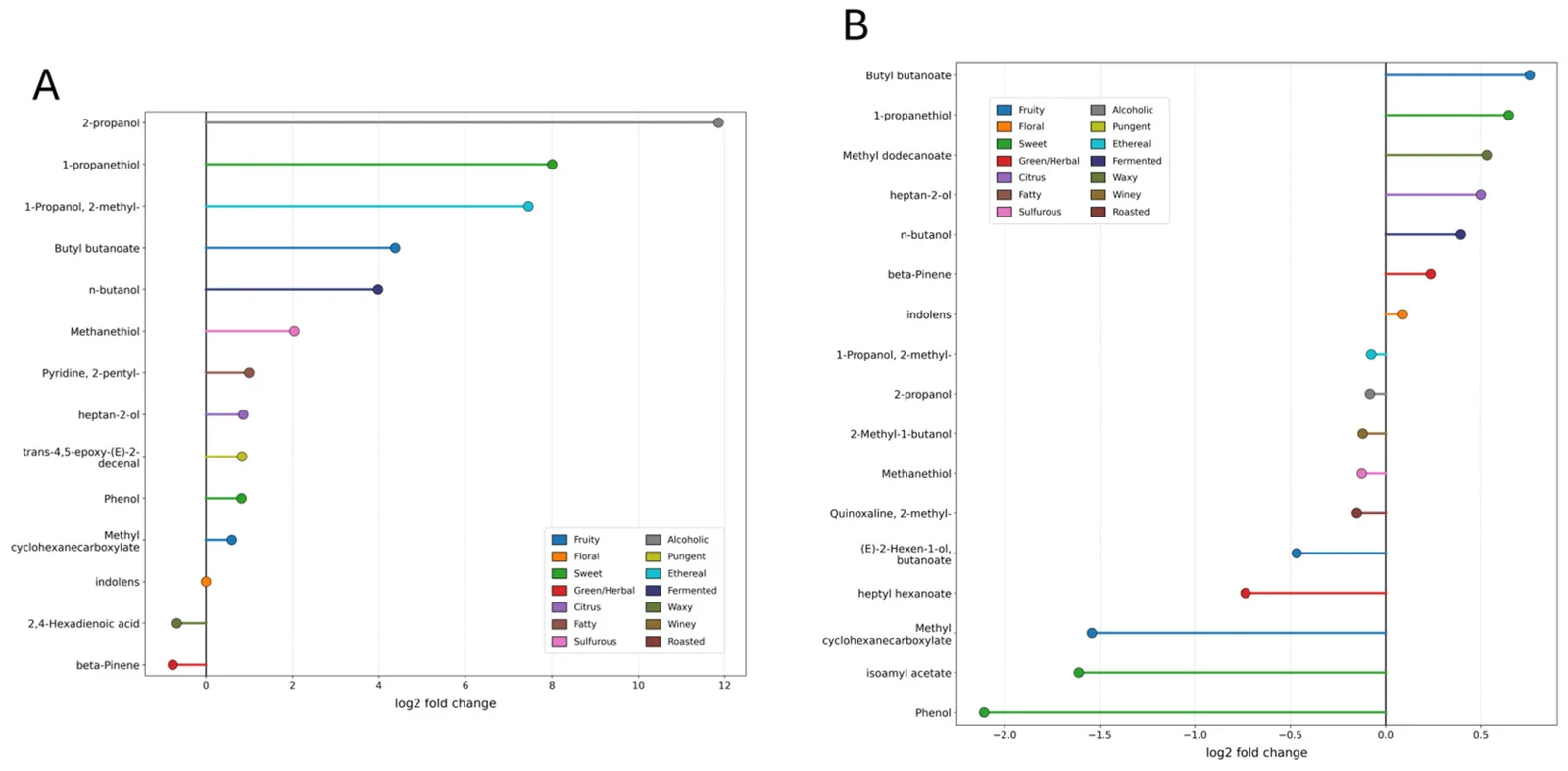
Pairwise modulation among persistent VOC pools using log2 fold change. (**A**) Log2 fold changes for compounds detected in both Pre and Post, showing fermentation-associated quantitative shifts. (**B**) Log2 fold changes for compounds detected in both Post and Aged, showing selective changes during aging. Positive values indicate higher abundance in the later stage of each comparison. Pre, pre-fermentation must; Post, post-fermentation mead; Aged, mead bottle-aged for 1 year.

To distinguish abundance modulation from identity turnover, pairwise comparisons were performed using log2 fold change restricted to VOCs detected in both conditions for each comparison (Figure 6). Fourteen VOCs were shared between Pre and Post. Within this persistent set, strong positive shifts dominated by alcohol and sulfur-containing compounds were measured in Post relative to Pre. The largest increases were observed for 2-propanol, which rose from 29.39 to 108,993.84 µg/L hexanal equivalents, and 1-propanol, 2-methyl-, which increased from 6.35 to 1113.32 µg/L hexanal equivalents. Sulfur-associated compounds also increased strongly, including 1-propanethiol from 11.32 to 2911.37 µg/L hexanal equivalents and methanethiol from 67.96 to 279.84 µg/L hexanal equivalents. Additional shared compounds, such as n-butanol, heptan-2-ol, butyl butanoate, methyl cyclohexanecarboxylate, phenol, and trans-4,5-epoxy-(E)-2-decenal, also shifted between Pre and Post, but with substantially smaller magnitudes (Figure 6A). These results indicate that fermentation drives the principal quantitative amplification within the persistent VOC pool.

Seventeen VOCs were shared between Post and Aged. Within this set, aging associated effects were smaller and more selective than the fermentation-associated shifts. Several compounds decreased during aging, including isoamyl acetate from 65.85 to 21.56 µg/L hexanal equivalents, phenol from 1.94 to 0.45 µg/L hexanal equivalents, methyl cyclohexanecarboxylate from 2.01 to 0.69 µg/L hexanal equivalents, and (E)-2-hexen-1-ol, butanoate from 3.36 to 2.43 µg/L hexanal equivalents. In contrast, 1-propanethiol increased further from 2911.37 to 4556.49 µg/L hexanal equivalents, butyl butanoate increased from 7.26 to 12.27 µg/L hexanal equivalents, heptan-2-ol increased from 1.09 to 1.54 µg/L hexanal equivalents, n-butanol increased from 33.24 to 43.66 µg/L hexanal equivalents, and methyl-dodecanoate increased from 0.09 to 0.13 µg/L hexanal equivalents. Major alcohols remained quantitatively dominant but changed only modestly, with 2-propanol decreasing from 108,993.84 to 102,917.73 µg/L hexanal equivalents and 1-propanol, 2-methyl- decreasing from 1113.32 to 1056.15 µg/L hexanal equivalents (Figure 6B). Together, these patterns support a two-step quantitative model in which fermentation establishes the main persistent VOC baseline, whereas aging acts primarily as a fine-tuning phase through selective modulation of ester, phenolic, sulfur, and a subset of alcohol associated features.

### 3.7. Stage-Resolved Mapping of Dominant VOCs to Descriptor-Linked Chemical Structure

**Figure 7:**
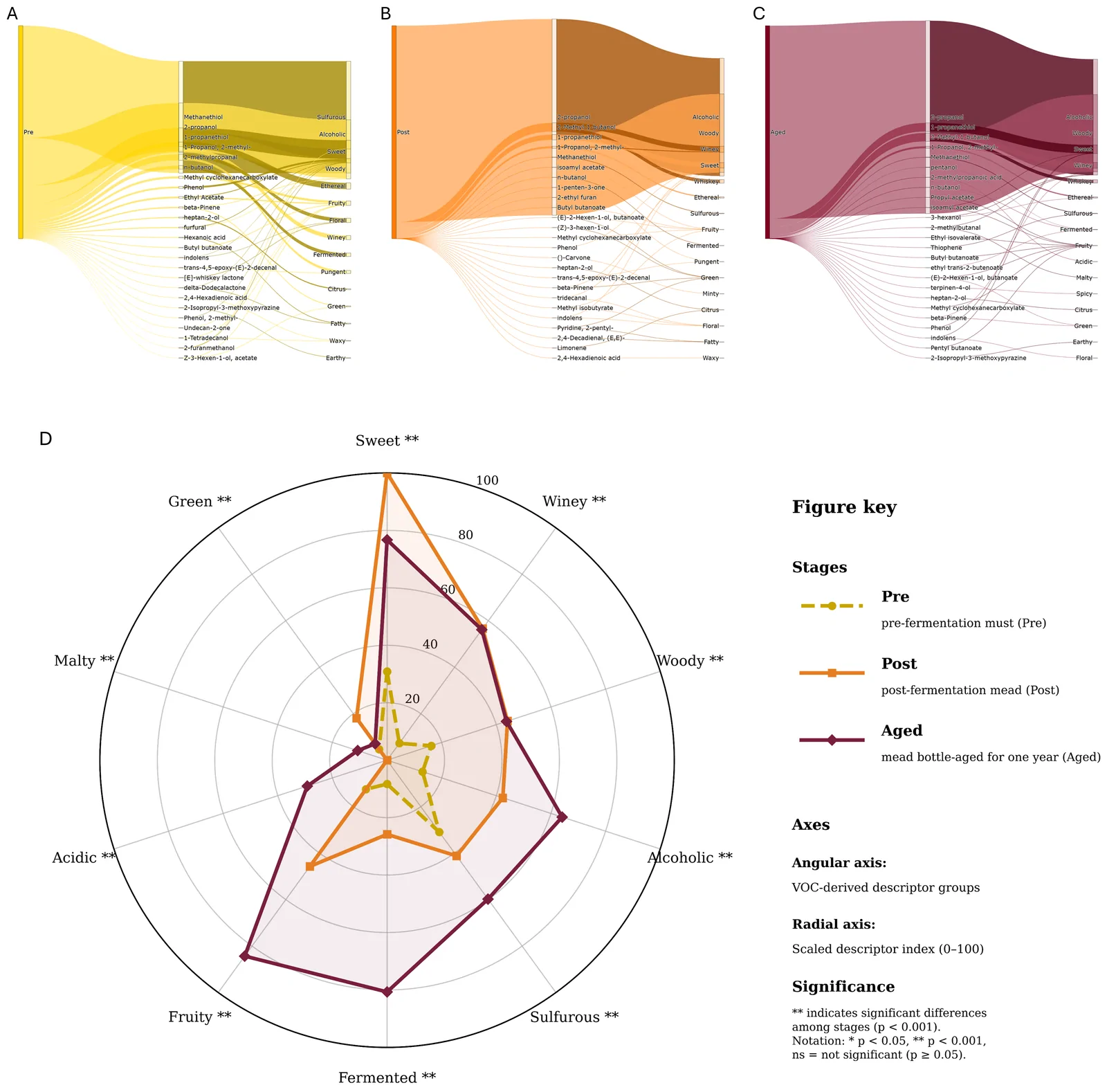
Stage-resolved mapping of dominant putative VOC candidates to database-derived descriptor categories. Sankey diagrams show the 25 most abundant putative VOC candidates in representative composite samples from (**A**) Pre, (**B**) Post, and (**C**) Aged stages. Link widths were scaled using log1p-transformed abundance values. (**D**) Radar plot summarizing the scaled descriptor-linked chemical index across stages. Pre, pre-fermentation honey must; Post, post-fermentation mead; Aged, mead bottle-aged for 1 year. These panels describe descriptor-linked chemical patterns and do not represent sensory-panel measurements. All compound names shown are putative electronic-nose database matches that were not confirmed with authentic standards or by GC-MS.

The 25 putative VOC candidates with the highest mean semi-quantitative abundance at each stage were mapped to their primary database-derived descriptor groups using Sankey diagrams (Figure 7A–C). Link widths were weighted using log1p-transformed abundance values to reduce dominance by a small number of highly abundant signals. This analysis summarizes descriptor-linked chemical structure and does not represent direct sensory intensity. The Pre profile was comparatively compact, with low-to-moderate flows towards sweet, fruity, floral, woody, green, fatty, and waxy categories (Figure 7A). Representative contributors included 2-methylpropanal, ethyl acetate, furfural, β-pinene, phenol, methanethiol, *n*-butanol, and methyl cyclohexanecarboxylate.

After fermentation, the Post profile expanded markedly, with stronger flows towards alcoholic, winey, whiskey, ethereal, sweet, green, pungent, woody, fruity, and sulfurous categories (Figure 7B). This pattern was driven mainly by 2-propanol, 2-methyl-1-butanol, 1-propanol, 2-methyl-, and 1-propanethiol, together with candidates such as 1-penten-3-one, 2-ethyl furan, (Z)-3-hexen-1-ol, carvone, isoamyl acetate, and butyl butanoate.

The Aged profile retained strong alcoholic and winey-linked signals but showed greater contributions from fermented, fruity, sulfurous, acidic, malty, coconut, earthy, and spicy categories (Figure 7C). Key contributors included pentanol, 2-methylpropanoic acid, 2-methylbutanal, thiophene, [Z]-whiskey lactone, terpinen-4-ol, propyl acetate, ethyl isovalerate, and 1-propanethiol.

Figure 7D summarizes these patterns as a scaled descriptor-linked chemical index. Compared with Pre, Post showed strong increases in sweet, winey, woody, alcoholic, and sulfurous indices. Aged retained these fermentation-associated features while showing greater fermented, fruity, acidic, and sulfurous contributions. Overall, the profile shifted from a diverse honey-must baseline to a fermentation-expanded chemical pattern and then to a selectively remodeled aged profile.

## 4. Discussion

### 4.1. Fermentation Was the Dominant Driver of Volatile Reorganization, While the Pre-Fermentation Profile Represented a Honey-Derived Baseline

The largest chemical transition in *T. diversifolia* mead occurred during fermentation rather than during the subsequent 12 months of storage. In the present dataset, glucose and fructose were depleted to near zero after fermentation and remained low thereafter, while the VOC profile changed much more strongly from Pre to Post than from Post to Aged. This pattern indicates that the aged product was not a chemically independent endpoint, but a selectively remodelled derivative of the fermentation established profile, which is consistent with mead studies showing that fermentation produces the main expansion and reweighting of volatile compounds before later maturation fine tunes the profile [1,3].

The design does not include a negative control. Because neither an uninoculated must incubated under fermentation conditions nor an unfermented honey stored in a bottle for the same period was analysed, the compounds detected only after fermentation cannot be attributed to yeast metabolism on the basis of this experiment alone. Throughout the following discussion, compounds are described by the stage at which they were detected, and the mechanisms proposed are drawn from the fermentation and beverage-aging literature rather than demonstrated here. The untargeted UHPLC-QTOF/MS profile resolved 10 non-volatile features whose detection differed across the three stages. Five features absent from the must were detected after fermentation, and two were detected only after 1 year of storage, a distribution consistent with the volatile data in showing fermentation as the larger transition and aging as a smaller, more selective one. The predominance of peptide and peptide-derivative features in the UHPLC-QTOF/MS profile suggests that nitrogen-containing metabolites represent an important component of the detected non-volatile chemical profile of the mead samples. The annotated features included peptides ranging from a dipeptide to a heptapeptide. These assignments are putative library-matched candidates rather than identifications, and the residue sequences given are not established by accurate mass alone, since every re-ordering of the same residues has an identical mass; they are discussed here at the level of compound class. Their occurrence may reflect the combined contribution of the original honey matrix and biochemical processes associated with fermentation. Amino acids and related nitrogen-containing metabolites serve important nutritional and metabolic functions during yeast fermentation and may also act as precursors for flavor-active metabolites. Consequently, changes in yeast growth, metabolism, and cellular turnover during fermentation may influence the formation, degradation, and detectability of peptide-related metabolites [15]. Fermentation and subsequent aging can also modify the chemical composition of mead, potentially altering the distribution of metabolites throughout the production process [15,16]. In addition to peptide-related features, the annotation of an aromatic heterocycle, a polyketide-derived quinone, a sulfated carbohydrate derivative, and two further heterocyclic candidates indicates a chemically diverse non-volatile fraction, although each of these annotations likewise remains putative. The stage-specific occurrence of these features may indicate changes in their formation, transformation, or detectability throughout mead production. Such changes during maturation may subsequently contribute to modifications in the chemical and sensory characteristics of the final beverage [16].

At the class level, fermentation caused a marked increase in alcohols and sulfur-containing compounds and a broader diversification of esters, indicating that yeast metabolism established the main volatile backbone of this mead. This interpretation is chemically plausible because alcoholic fermentation generates higher alcohols and related metabolites through glycolytic carbon flow and amino acid catabolism, while ester formation depends on the interaction of alcohols with acyl donors under yeast enzymatic control [2,7]. Accordingly, the strong post-fermentation emergence of compounds such as 2-propanol, 1-propanol, 2-methyl-, and 2-methyl-1-butanol is consistent with a fermentation-constructed aroma system, although the absolute values of some highly abundant analytes should still be interpreted cautiously until analytically validated. The increase in branched-chain higher-alcohol candidates, including 2-methyl-1-butanol, is consistent with amino-acid catabolism through the Ehrlich pathway, involving transamination, decarboxylation, and reduction of amino-acid-derived intermediates. Methanethiol may arise from yeast methionine catabolism, whereas other detected thiol candidates may result from secondary transformations within the sulfur-metabolism network. These mechanistic interpretations remain provisional because the electronic-nose compound assignments were not confirmed using authentic standards.

The identity-based analyses support the same conclusion. The greatest compositional discontinuity occurred between the pre-fermentation must and the two later stages, whereas Post and Aged shared a substantially larger overlap. This indicates that fermentation did not simply modulate the abundance of an inherited honey volatilome but reset a major part of the detectable chemical inventory. A comparable restructuring was reported by Chitarrini et al., who found clear differences between pre-fermentation and fermented mead samples, with post-fermentation products showing a broader and reweighted pool of alcohols, acids, esters, and terpenes [3]. An important strength of the present design is that it shows the Pre sample to be more than a chemically weak precursor. Instead, it represents a distinctive honey-derived baseline. In this study, Pre-enriched compounds such as furfural, 2-methylpropanal, hexanoic acid, [E]-whiskey lactone, delta-dodecalactone, 2-furanmethanol, and beta-pinene plausibly reflect botanical, handling, or storage-related features of the tree marigold honey matrix before strong yeast remodelling occurred. This interpretation agrees with the broader honey volatile literature, which shows that honey contains a diverse and origin-dependent pool of alcohols, aldehydes, ketones, acids, esters, furans, terpenes, sulfur compounds, and other minor constituents, with several hundred compounds reported across honey types [17].

The decline or disappearance of many Pre-associated compounds after fermentation can be explained by several non-exclusive mechanisms. Native honey compounds may become less prominent through dilution, volatilization, or matrix-partitioning effects, and they may also be transformed directly or indirectly by yeast metabolism. At the same time, large quantities of newly formed fermentation products may dominate the post-fermentation signal and effectively obscure lower abundance-inherited compounds at the whole profile level. This kind of substrate overwriting is biologically reasonable in mead because honey must is nutritionally restrictive, and low assimilable nitrogen is repeatedly linked to sluggish fermentation and altered volatile output; under such conditions, yeast strain and nitrogen regime can substantially shift the balance of higher alcohols, esters, fatty acids, and other aroma-relevant metabolites [18,19]. These results support a process centred interpretation in which the pre-fermentation volatilome functions as a chemically diverse honey derived baseline, while fermentation acts as the dominant phase of volatile construction and compositional reset. Aging then modifies that fermentation established backbone through selective persistence, gain, and loss rather than restoration of the original honey pattern.

### 4.2. Interpretation of the Differences Between the Post and Aged Composites

In contrast to the larger Pre–Post difference, the Post–Aged contrast was more selective. In the present dataset, Post and Aged remained more similar to each other than either was to Pre, indicating that the aged composite retained much of the composition measured after fermentation rather than presenting a wholly new profile. This interpretation is consistent with recent mead work showing that 1 year of aging changes aroma composition while still preserving strong dependence on the chemistry established during fermentation [4].

The most distinctive aging-associated change in this study was the selective enrichment of the carboxylic acid fraction, particularly the appearance of 2-methylpropanoic acid in Aged, together with a lower pH relative to Post. Such changes are consistent with net acidifying and re-equilibration processes during storage, since bottle aging is known to involve slow oxidation, hydrolysis, esterification, and related matrix-dependent reactions that progressively reshape volatile composition rather than simply causing uniform aroma loss [20,21]. The storage temperature of 24–28 °C is warmer and less stable than conventional cellar conditions, and the rates of the hydrolytic, esterification, and oxidative reactions that occur during bottle aging are temperature-dependent. The changes reported here should therefore be understood as the outcome of 1 year of warm ambient storage and not as a general description of mead maturation. In addition, the aged sample contained several compounds absent from Post, including pentanol, propyl acetate, ethyl isovalerate, ethyl trans-2-butenoate, pentyl butanoate, 2-methylbutanal, 2-heptenal, thiophene, terpinen-4-ol, and [Z]-whiskey lactone, indicating that prolonged storage generated a distinct maturation associated layer composed of acid, aldehyde, ester, sulfur, terpene, and lactone-related features.

This selective remodelling is also mechanistically consistent with dynamic ester chemistry during bottle aging. Esters do not necessarily decline in a uniform way during storage. Instead, their levels reflect the competing effects of hydrolysis and re-esterification, which depend on precursor availability, ethanol concentration, acidity, and matrix composition; recent kinetic work has shown that ester evolution during aging is governed by equilibrium reactions rather than by one-way degradation alone [22]. This framework matches the present data well: some post-fermentation esters decreased during aging, such as isoamyl acetate and methyl cyclohexanecarboxylate, whereas others appeared or increased, including propyl acetate, ethyl isovalerate, butyl butanoate, and methyl dodecanoate. The aged profile therefore appears to reflect dynamic re-equilibration among ester pools rather than universal ester loss.

The appearance of 2-methylbutanal and 2-heptenal further supports the involvement of aldehyde chemistry during maturation because aldehydes are well-known products of oxidation-related and Strecker-type pathways during beverage aging [23]. Likewise, the persistence and differentiation of sulfur-linked compounds in Aged are chemically plausible, since sulfur species and their precursors may remain reactive during storage and continue to contribute to bottle-aged aroma evolution [20]. Taken together, these observations are compatible with a more selective secondary difference superimposed on the composition measured after fermentation, with higher acid-associated responses and a limited number of additional candidates, rather than a wholly reconstructed profile.

### 4.3. Honey-Based Substrate Chemistry and the Renewed Innovation Landscape in Modern Mead Brewing

Mead fermentation begins from a substrate that is chemically distinct from grape or fruit must, and this distinction is central to interpreting the volatile trajectories observed across Pre, Post, and Aged stages in the present study. Honey contributes a complex and highly variable background of native volatile and semi-volatile compounds whose composition depends strongly on botanical and geographical origin, post-harvest handling, and analytical method. Reviews of honey aroma chemistry report that monofloral and multifloral honeys contain broad classes of compounds, including alcohols, aldehydes, ketones, esters, carboxylic acids, terpenoids, benzene derivatives, phenolic compounds, furans, furanones, and nitrogen-containing compounds, with some surveys reporting more than 600 volatile constituents across honey types [24]. Representative compounds repeatedly reported in honeys from different botanical origins include benzaldehyde, furfural, 2 phenylethanol, linalool-derived terpenoids, acetic acid, and other short-to-medium chain-oxygenated compounds. This inherited chemical background provides a plausible basis for the stage-specific Pre candidates detected in our dataset and helps explain why several compounds are present before fermentation but become quantitatively less prominent after yeast-derived metabolites dominate the profile [25,26,27].

The importance of this honey-derived baseline is amplified by the fact that honey must is not only compositionally different from fruit must, but also nutritionally more challenging for yeast. Mead literature consistently identifies low yeast assimilable nitrogen, limited micronutrient availability, low buffering capacity, and the highly concentrated sugar environment of honey must as major factors contributing to slow, incomplete, or stuck fermentations [26]. These constraints do not merely affect fermentation kinetics. They also influence how yeast partitions carbon and nitrogen into biomass, ethanol, higher alcohols, esters, sulfur compounds, and other aroma-relevant metabolites. In this context, the marked shift from a chemically diverse but comparatively low-abundance Pre profile to an alcohol and sulfur-enriched Post profile in our data is mechanistically consistent with a fermentation system in which yeast metabolism is building a new aroma architecture under substrate specific nutritional and osmotic constraints rather than simply modifying fruit like precursors [12,19,28,29].

In the last decade, mead research and practice have also shifted from traditional single-yeast, single-nutrient approaches toward a more innovation-driven brewing paradigm aimed at improving consistency, aroma quality, and product differentiation. Contemporary peer-reviewed syntheses emphasize optimization of yeast-assimilable nitrogen in honey must, measurement and regulation of nitrogen additions, and careful selection of yeasts with appropriate nitrogen demand and aroma outputs as central strategies for modern mead production [30]. In parallel, recent experimental studies on specialty honeys demonstrate that adjusting starting nitrogen levels and combining nutrient and micronutrient supplementation can increase fermentation efficiency and reshape VOC composition toward more desirable fruity ester profiles while reducing less desirable higher alcohol and fatty acid-related notes, with additional modulation by yeast strain and interaction effects between nitrogen and strain [12]. Innovation is also expanding beyond *Saccharomyces cerevisiae*. Recent work exploring non-Saccharomyces yeasts and enzymatic traits such as beta glucosidase activity illustrates a growing interest in unlocking bound aroma precursors and diversifying volatile outputs, although such approaches must balance fermentation completeness and ethanol yield against aroma novelty [31]. At the field level, bibliometric synthesis of mead aroma research similarly indicates increased attention to the roles of esters, alcohols, acids, phenols, and terpenes, and it highlights fermentation conditions and yeast metabolism as dominant determinants of aroma profiles, which indicates that stage-resolved chemical profiling of mead is aligned with current research directions in the field [5].

Taken together, the available evidence supports a process-level interpretation in which honey provides a chemically diverse but variable starting volatilome, fermentation imposes the dominant metabolically driven restructuring, and aging acts as a subsequent filtering and rebalancing phase. Within that framework, the Pre-unique compounds observed here are best viewed as honey-inherited or handling-associated candidates, the Post-unique compounds as fermentation-constructed products generated under honey specific nutritional constraints, and the Aged-unique compounds as outcomes of selective persistence and slower storage chemistry. This perspective is especially useful for mead because it supports candidate development and interpretation in a substrate specific way rather than transferring assumptions directly from grape wine systems, even though shared principles such as ester formation during fermentation and ester re-equilibration during aging still apply.

### 4.4. Relevance of Stage-Resolved Volatile Profiling for Mead Quality Assessment and Maturation Monitoring

An important contribution of the present study is that it moves mead aroma research from endpoint description toward stage-resolved process interpretation. Recent reviews emphasize that mead-quality evaluation still lacks sufficiently integrated analytical frameworks linking honey origin, fermentation behaviour, volatile evolution, and sensory outcome across the full production chain, rather than only at the finished product stage [5]. Within that context, the present work provides a chemically structured model in which the Pre profile represents the incoming honey baseline, the Post profile captures fermentation driven volatile construction, and the Aged profile reflects selective maturation-related divergence. This is useful because it converts a descriptive three-stage dataset into an interpretable framework for production monitoring. From an applied perspective, this framework has potential value for raw material characterization, fermentation tracking, and maturation assessment. Honey VOC profiles are already widely recognized as useful indicators of botanical origin and product differentiation, and chemometric analysis of honey volatiles has been shown to classify honeys with high accuracy across floral sources [32,33]. In mead, where fermentation subsequently restructures that inherited signal, stage-resolved profiling adds an extra level of information by showing not only what the starting matrix was, but also how much of that matrix was retained, overwritten, or re-expressed during fermentation and aging. In practical terms, this means that Pre-associated compounds may be useful for evaluating the incoming honey substrate, whereas Post- and Aged-associated compounds may be more relevant for judging fermentation expression and maturation status.

The value of this approach is strengthened by the growing diversification of the mead sector. Modern mead production increasingly involves specialty honeys, tailored nutrient regimes, selected yeast strains, alternative fermentation strategies, and different maturation conditions, all of which may alter volatile composition and sensory properties [4]. Under these conditions, a stage-resolved chemical framework is more informative than judging quality only from the final product because it can help distinguish whether an unusual aroma outcome originated primarily from the honey substrate, from fermentation performance, or from storage-driven transformation. This is particularly relevant for premium and region-specific meads, where product identity may depend on both preservation of honey character and controlled development of maturation-related complexity.

At the same time, the practical use of these compounds should currently be framed as candidate chemical markers, not validated sensory markers. Mead aroma cannot be inferred from concentration data alone, because odor thresholds, matrix effects, and perceptual interactions can strongly modify the sensory contribution of individual compounds [2,34]. This point is especially important here because the present analytical workflow recovered an extracted volatile and semi-volatile fraction rather than a full headspace aroma profile. Recent methodological reviews likewise stress that analytical platform choice strongly influences which compounds are detected and how directly they can be linked to sensory evaluation in mead. A second important limitation is that the proposed candidate-based approach has been developed here from a single honey type under one fermentation and one bottle-aging condition. The observations are limited to one batch of *T. diversifolia* mead under the present conditions [13] and should not be treated as supported for other systems. Establishing whether the pattern described here reflects a general feature of mead maturation will require a design that this study did not have: independent fermentations analysed separately rather than pooled, sampling at intermediate points such as 1, 3, 6, and 12 months, an unfermented and an uninoculated control held under the same storage conditions, orthogonal confirmation of volatile identities by GC-MS with authentic standards, targeted quantification of the candidate compounds, and sensory evaluation [1]. The candidate features reported here are intended to define the target list for such a study.

Taken together, the broader significance of this work lies in showing that stage-resolved volatile profiling may do more than catalogue compound changes. It can help define where aroma identity originates, when the major restructuring of that identity occurs, and how later storage modifies an already established volatile backbone. For mead science, this provides a clearer route toward substrate-aware quality assessment and maturation monitoring. For mead production, it offers a basis for moving from descriptive post-hoc analysis toward more systematic, candidate-guided process control.

### 4.5. Limitations

Several limitations constrain how these results should be read. First, the volatile and non-volatile datasets are not biologically replicated. Equal aliquots from the three fermentation vessels were combined into one composite sample per stage before electronic-nose and UHPLC-QTOF/MS analysis, and the replicate injections performed on those composites measure instrument repeatability rather than variation between fermentations. Variability among vessels therefore cannot be estimated, no measure of dispersion can be attached to any stage, and no difference between stages can be tested for significance. Only the physicochemical measurements, which were obtained from three independent production replicates, support statistical inference. For the same reason, principal component analysis was not applied to the three composite profiles: with three mean-centred observations, the first two components necessarily account for all of the variance, so any apparent separation would follow from the number of samples rather than from a difference between stages.

Second, the design includes no negative control. Neither an unfermented honey held in a bottle for the same period nor an uninoculated must incubated under fermentation conditions was analysed. It is therefore not possible to separate compounds generated by yeast metabolism from compounds intrinsic to the honey, from slow chemical change during storage, or from oxygen ingress through the closure. Where a mechanism is mentioned in the Discussion, it is identified as a literature-based possibility rather than a mechanism demonstrated by this design, and the pre-fermentation must serves as a starting-material baseline rather than as a negative or time-matched control.

Third, the compound assignments are tentative. Volatile candidates were matched against the AroChemBase library using ultra-fast gas chromatographic-retention behaviour with a match score of at least 80% and were not confirmed with authentic standards or by GC-MS. Abundances are semi-quantitative responses expressed as hexanal equivalents; because flame-ionisation response factors and headspace partition coefficients differ between chemical classes, these values compare detector signals rather than concentrations, and class-level sums are indicative only. The non-volatile annotations are likewise putative, reach at most level 2 of the Schymanski scale, and were obtained without a pooled quality-control sample in the analytical sequence.

Fourth, bottle aging was carried out at ambient temperature between 24 and 28 °C. This reflects the storage conditions typical of artisanal production in Northern Thailand, but it is warmer and less stable than cellar storage, and the rates of hydrolytic, esterification, and oxidative reactions are temperature-dependent. The aged profile reported here should not be extrapolated to temperature-controlled storage. Headspace oxygen was not measured. Fifth, the storage period is bracketed by only two sampling points. Sampling at the end of fermentation and after 12 months describes the difference between two endpoints and does not resolve when any change occurred, so no maturation kinetics can be inferred.

Sixth, because the three stages were sampled at different times, the duration of frozen storage before analysis differed between them: the pre-fermentation and post-fermentation aliquots were held at −80 °C longer than the aged aliquot. The three composites were prepared on the same day and acquired within a single instrumental sequence in order to minimise inter-run analytical variation, but this does not correct the unequal storage periods. Sampling point and frozen-storage history are therefore inseparable in this design, and because no matrix-specific stability test and no time-matched storage control were included, the contribution of frozen storage can be neither quantified nor excluded. The absence of a pooled quality-control sample further limits the assessment of analytical drift; the replicate injections used in its place measure repeatability within the sequence but do not detect systematic drift across it. The interval before analysis reflects a deliberate choice between two competing sources of error. Analysing the stages in separate instrumental batches would have introduced a between-batch effect, which in untargeted LC-MS affects signal intensity, mass accuracy, and retention time, and it is difficult to correct without pooled quality-control samples distributed across batches [35,36]; three composite samples such a correction could not be implemented meaningfully. Metabolite stability at −80 °C over an interval of this length is, by comparison, comparatively well characterised, with only a small proportion of metabolites reported to change significantly over the 1st years of storage [37,38]. On that basis, the three composites were prepared on the same day and acquired in a single uninterrupted sequence, accepting unequal frozen storage in order to remove the batch effect.

Finally, the study covers one monofloral honey, one commercial *S. cerevisiae* strain, one fermentation regime, and one storage condition in a single production batch. The findings describe *T. diversifolia* mead produced and stored in this way. Determining whether the same stage-resolved organisation applies to other floral sources, yeast strains, nutrient regimes, and storage temperatures will require a replicated, multi-factor design with time-course sampling, orthogonal GC-MS confirmation, targeted quantification, and sensory evaluation.

Completion of fermentation was established from the cessation of visible carbon dioxide evolution and progressive clarification rather than from a numerical endpoint. Final specific gravity, ethanol concentration, and titratable acidity were not determined, so the extent of sugar-to-ethanol conversion is characterised only indirectly by the depletion of glucose and fructose below the limit of quantification reported in Table 1.

The non-volatile annotations reported in Table 2 are putative. Retention time, measured accurate mass, and the stage at which each feature was detected are direct measurements, and every candidate formula satisfies the ≤10 ppm mass-accuracy criterion, but no annotation was confirmed against an authentic standard and none therefore exceeds level 2 of the Schymanski scale. Two further constraints should be borne in mind. More than one molecular formula, and, in each case, several isomeric structures, are consistent with a given measured mass at this level of mass accuracy, so the entries in Table 2 represent the highest-ranked candidates returned by library matching rather than established identities. For the peptide-related features, the elemental composition constrains the residue content but not the sequence, because all re-orderings of the same residues are isobaric. The compound names should accordingly be read as candidates. No interpretation offered in this manuscript depends on the identity of any individual feature, and confirmation will require MS/MS acquisition against authentic standards or a curated reference spectral library.

## 5. Conclusions

In this single-batch study of *Tithonia diversifolia* honey mead, biologically replicated physicochemical measurements showed depletion of glucose and fructose below the limit of quantification during fermentation and a small decrease in pH during 1 year of ambient bottle aging at 24–28 °C. Descriptive profiling of one composite sample per stage showed that the volatile composition differed considerably more between the pre-fermentation must and the fermented mead than between the fermented and the aged mead. The post-fermentation profile was dominated by alcohol- and sulfur-associated responses, and the aged sample additionally contained acid-, ester-, aldehyde-, and sulfur-associated candidates, including 2-methylpropanoic acid, pentanol, propyl acetate, 2-methylbutanal, thiophene, and terpinen-4-ol. These observations are consistent with fermentation establishing most of the volatile composition and 1 year of ambient bottle aging being associated with a smaller, more selective difference. They describe one production batch of one monofloral honey and are not evidence that this pattern extends to other honeys, yeast strains, or storage conditions. The candidate features reported here define a focused target list for the replicated, time-resolved, and sensory-validated studies that would be required before any of them could be used to monitor maturation in *T. diversifolia* mead.

## Figures and Tables

**Table 1:** Physicochemical characteristics of *T. diversifolia honey* mead across Pre, Post, and Aged stages.

Parameter	Pre	Post	Aged
pH	3.80 ± 0.07 ^ab^	3.88 ± 0.05 ^a^	3.67 ± 0.05 ^b^
Colour	White	Extra white	Water white
Glucose (mg/mL ± SD)	71.9 ± 0.2	<1 mg/mL	<1 mg/mL
Fructose (mg/mL ± SD)	86.6 ± 0.8	<1 mg/mL	<1 mg/mL

Values are mean ± SD from three biologically independent production replicates. Different superscript letters indicate significant differences among stages at *p* < 0.05. <1 mg/mL indicates a value below the lowest calibration level, which is the validated limit of quantification. Colour is reported as the USDA colour designation, which corresponds to defined ranges on the Pfund scale (water white, ≤8 mm; extra white, >8–17 mm; white, >17–34 mm); the sequence observed here therefore represents a progressive shift towards the lighter end of the scale.

## Data Availability

The data presented in this study are included in the article. Further inquiries can be directed to the corresponding authors.
